# Antimicrobial use patterns during the COVID-19 pandemic at an academic medical center

**DOI:** 10.1017/ash.2022.97

**Published:** 2022-05-16

**Authors:** Jacob Pierce, Erin Deja, Kimberly Lee, Michelle Doll, Michael Stevens

## Abstract

**Background:** The COVID-19 pandemic has made a significant impact on antimicrobial use patterns across health systems. We have described antibiotic use patterns at an academic medical center in Richmond, Virginia, before and after the onset of COVID-19. We also examined the impact on the proportional consumption of carbapenems (PoCC) metric. PoCC represents meropenem utilization relative to the narrower-spectrum antipseudomonal agents cefepime and piperacillin-tazobactam. Our institution practices antimicrobial restriction for meropenem. All other antibiotics included in the study data can be freely ordered by any provider. **Methods:** We evaluated antimicrobial use data from September 2018 through August 2021 using days of therapy (DOT) per 1,000 patient days. We included 18 months of data before and after the first recorded COVID-19 admission at our institution in March 2020. Mean comparisons were performed using the Welch 2-sample *t* test. The Bonferonni correction for multiple comparisons was utilized to determine significance with an initial baseline α of 0.05. All data analyses were performed using R software (R Foundation for Statistical Computing, Vienna, Austria, 2021). **Results:** Normality was evaluated with QQ-plots and all data demonstrated normality. Bonferroni correction produced an adjusted α value of 0.007. We detected significant increases in the use of cefepime, piperacillin-tazobactam, ceftriaxone, and azithromycin following the onset of the COVID-19 pandemic. We noted a significant decrease in the PoCC metric during this period. No significant change was noted for levofloxacin or meropenem. **Conclusions:** The COVID-19 pandemic produced significant changes in antimicrobial use patterns at our institution. We noted statistically significant increases in bacterial community-acquired pneumonia-focused antibiotics (ceftriaxone and azithromycin). We observed significant increases for cefepime and piperacillin-tazobactam. Interestingly, relative utilization of carbapenems as measured by the PoCC metric continued to decrease during this time. This trend was primarily driven by increases in cefepime and piperacillin-tazobactam utilization while meropenem utilization remained relatively constant. This study highlights the importance of looking at normalized antibiotic consumption data and not relative-use data alone.

**Funding:** None

**Disclosures:** None

